# Performance and considerations in the use of diagnostic mutation panels for clonality testing in non-small-cell lung carcinoma

**DOI:** 10.1016/j.esmoop.2025.105072

**Published:** 2025-05-14

**Authors:** J. Janssen, B. Andrade Barbosa, J.C. Machado, P. Hofman, Y. Kim, B. Ylstra, T. Radonic

**Affiliations:** 1 Department of Pathology, Cancer Center Amsterdam, Amsterdam UMC, Vrije Universiteit Amsterdam, Amsterdam, The Netherlands; 2 Institute of Molecular Pathology and Immunology of the University of Porto (IPATIMUP), Porto, France; 3 Institute for Research and Innovation in Health (i3S), Porto, Portugal; 4 Faculty of Medicine of the University of Porto, Porto, Portugal; 5 Clinical and Molecular Pathology Department, IHU RespirERA, Nice, France

**Keywords:** multiple pulmonary tumors, diagnostic test accuracy (DTA), next-generation sequencing (NGS), whole exome sequencing (WES)

## Abstract

**Introduction:**

Next-generation sequencing (NGS) mutation panels are widely implemented and commonly applied to aid clonality classification for non-small-cell lung carcinoma (NSCLC) patients with multiple tumors. Performance of different NGS panels for clonality classification, however, remains unresolved.

**Methods:**

We assembled 210 primary and metastatic pairs from lung adenocarcinoma (LUAD) and lung squamous-cell carcinomas (LUSC) of the TRACERx421 cohort for which gold standard clonality was confirmed by whole exome sequencing. We used four NGS panels ranging from 12 to 523 genes to determine clonality using the 2024 International Association for the Study of Lung Cancer (IASLC) recommendations.

**Results:**

With an oncogene panel, 30% LUAD and 74% LUSC pairs remained inconclusive with, respectively, 2% and 0% misclassified. Addition of tumor suppressor genes results in 5% LUAD and 5% LUSC inconclusive and, respectively, 2% and 1% misclassified. For large panels, 0%-1% was inconclusive and 1% misclassified for both LUAD and LUSC. Misclassifications occurred due to discordant *KRAS* mutations in clonal pairs or coincidentally shared *PIK3CA* mutations in non-clonal pairs.

**Conclusion:**

Oncogene panels result in many inconclusive results, most of which can be resolved by adding tumor suppressor genes. Notwithstanding, 1%-2% of patients remain challenging. Large NGS panels detect mutations in more genes than the IASLC recommendations, allowing definitive clonality classification.

## Highlights

• NGS mutation panels are widely implemented and used for clonality testing in NSCLC according to a 2024 IASLC algorithm.
• Oncogene-only panels result in many inconclusive results, most of which can be resolved by adding tumor suppressor genes.
• Some 1%-2% of patients remain challenging with any NGS panel.
• Based on our findings, we recommend use of large NGS panels and a minor adaptation of the 2024 IASLC algorithm.

## Graphical abstract

**Figure:**
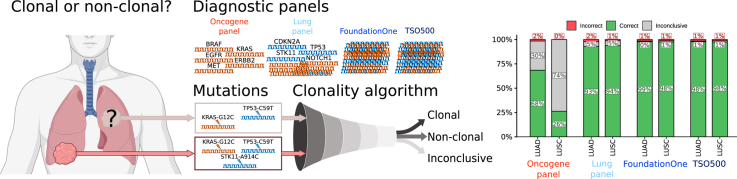

## Introduction

Diagnosis of multiple non-small-cell lung carcinomas (NSCLCs) as either separate primary lung cancers (SPLCs) or metastasis can significantly impact patient outcomes and treatment decision-making, notably for targeted therapies. Approaches available in routine clinical practice to assess the clonal relationship between multiple tumors historically included clinical^1^ and histological^2^ evaluations. Histological evaluation is widely available across pathology laboratories and was considered the default approach for clonality assessment. The advent, however, of molecular methods, e.g. large next-generation sequencing (NGS) mutation panels and whole exome sequencing (WES) in recent years has provided a reference to evaluate historical approaches, revealing that histological comparison of tumors alone is inaccurate in 20%-44% of pairs.^3^^,^^4^

Molecular analysis using targeted NGS panels designed for treatment guidance is available in many pathology laboratories, and therefore intuitively applied to aid in clonality testing.^5^ To accommodate the use of these NGS panels, decision trees to standardize the evaluation for clonality testing are proposed in the 2024 recommendation of the International Association for the Study of Lung Cancer (IASLC).^6^ Neither the percentage of patients for whom a final diagnosis can be determined, however, nor the test accuracy of different targeted NGS panels with such classification algorithms have been systematically determined.

In this short communication, we provide an objective evaluation of clonality classification using several common NGS panels combined with the recently proposed 2024 IASLC molecular algorithm^6^ in a dataset of tumor pairs,^7^^,^^8^ for which the clonality status was determined by WES as a gold standard reference.

## Materials and methods

### Gold standard clonal and non-clonal sample pair assembly

**Figure 1:**
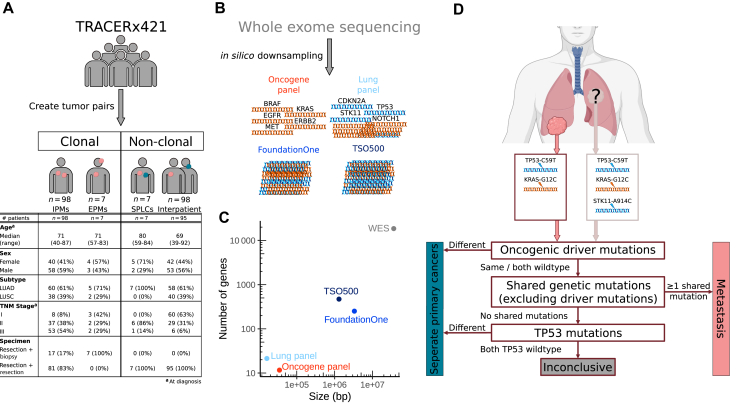
**Methodological overview**. (A) Assembly of WES mutation verified gold standard clonal and non-clonal sample pairs from the TRACERx421 cohort. The table displays the clinical characteristics of the tumor pairs. (B) Schematic illustration of *in silico* downsampling of WES data to four NGS panels. Tumor suppressor genes are indicated in blue and oncogenes are indicated in orange. (C) Base pair size (*x*-axis) and number of genes (*y*-axis) of WES and the four different NGS panels. Both axes are on a log-scale. (D) Schematic illustration of clonality assessment using the 2024 IASLC molecular classification algorithm^6^ (scheme adapted from Yang et al.^9^). EPM, extrapulmonary metastasis; IASLC, International Association for the Study of Lung Cancer; IPM, intrapulmonary metastasis; LUAD, lung adenocarcinoma; LUSC, lung squamous cell carcinoma; SPLC, separate primary lung cancer; TNM, tumor–node–metastasis; WES, whole exome sequencing. ^a^At diagnosis.

From the TRACERx421 cohort^7^^,^^8^ we assembled a total of 210 tumor pairs, each gold standard verified as either clonal or non-clonal using the WES mutation data based on the number of shared mutations (Figure 1A, Supplementary Table S1, available at https://doi.org/10.1016/j.esmoop.2025.105072). The WES sequence data derived from deep NGS of fresh frozen tumor material with matched normal material (with a median depth of 400x for both tumor and normal) which was subjected to state of the art NGS mutation calling algorithms that include extensive quality control steps were used as published.^7^^,^^8^ To create clonal pairs, we selected WES mutation data for all primary and intrapulmonary metastatic pairs available [lung adenocarcinoma (LUAD); *N* = 60, lung squamous-cell carcinoma (LUSC); *N* = 38] which were supplemented with primary and extrapulmonary metastatic pairs (LUAD; *N* = 5, LUSC; *N* = 2). For the non-clonal pairs, we selected all available SPLC pairs (LUAD; *N* = 7, LUSC; *N* = 0) which were supplemented with randomly selected interpatient primary tumor pairs to match the number of clonal pairs (LUAD; *N* = 58, LUSC; *N* = 40). The clinical characteristics of all tumor pairs are presented in Figure 1A.

### Test accuracy calculations for four NGS mutation panels

Calls for four common NGS mutation panels were extracted from the WES mutation calls *in silico* by downsampling the overlapping regions (Figure 1B). We selected two small panels: an oncogene panel (0.014 Mb, 12 oncogenes) and an NSCLC-specific panel (0.034 Mb, 27 genes including tumor suppressors), and two large panels: FoundationOneCDx (3.27 Mb, 324 genes) and Illumina’s TruSight Oncology 500 (TSO500) (1.28 Mb, 523 genes) (Figure 1C, Supplementary Table S2, available at https://doi.org/10.1016/j.esmoop.2025.105072). With each of the four NGS panels, clonal, non-clonal or inconclusive relation was determined for all sample pairs using the clonality algorithm according to 2024 IASLC recommendations^6^ (Figure 1D).

Sensitivity, specificity and diagnostic test accuracy (DTA) were calculated for each panel using all 65 clonal and 65 non-clonal LUAD pairs and all 40 clonal and 40 non-clonal LUSC pairs as verified by the WES gold standard. For details, see Supplementary Methods, available at https://doi.org/10.1016/j.esmoop.2025.105072.

## Results

### Performance of clonality assessment using four NGS mutation panels with WES as gold standard

**Figure 2:**
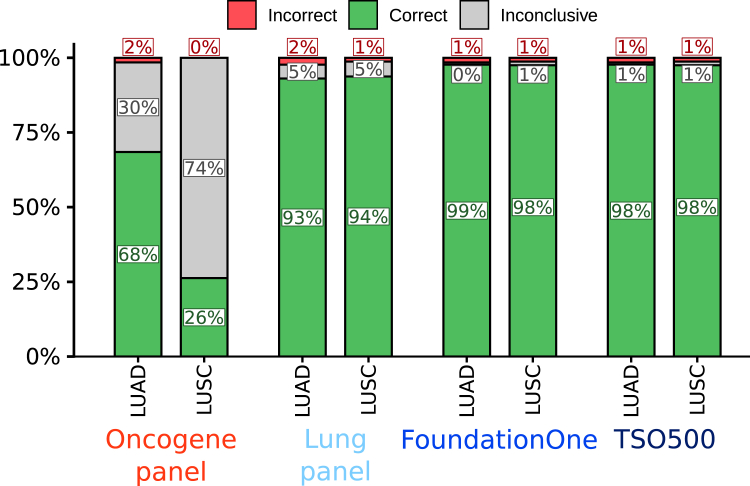
Accuracy of diagnostic NGS mutation panels to test clonality of multiple lung tumors based on 65 clonal and 65 non-clonal LUAD pairs and 40 clonal and 40 non-clonal LUSC pairs of the TRACERx421 cohort. LUAD, lung adenocarcinoma; LUSC, lung squamous cell carcinoma.

The oncogene panel yielded a high number of inconclusive pairs, with 30% of the LUAD and 73.8% of the LUSC pairs unresolved (Figure 2, Supplementary Table S3, available at https://doi.org/10.1016/j.esmoop.2025.105072). Inconclusive results were due to lack of any detected mutations in one or both tumors (LUAD; 12%, LUSC; 71%) or only a matching driver mutation (LUAD; 18%, LUSC; 3%). Nevertheless, it showed high sensitivity and specificity with 1.5% and 0% misdiagnosed pairs for LUAD and LUSC, respectively, which comes with a DTA of 98.5% and 100%. With only 15 extra genes, the NSCLC-specific panel reduced the number of inconclusive pairs, to 4.6% for LUAD and 5% for LUSC. This reduction was caused both due to less inconclusive results by lack of any detected mutation (LUAD; 2%, LUSC; 5%) and by only a matching driver mutation (LUAD; 3%, LUSC; 0%). The number of misdiagnosed pairs slightly increased to 2.3% for LUAD and 1.3% for LUSC, which comes with a DTA of 97.7% and 98.7% (Figure 2, Supplementary Table S3, available at https://doi.org/10.1016/j.esmoop.2025.105072). For FoundationOne and TSO500, 98%-99% of samples can be correctly classified, with only 0%-1% inconclusive pairs, but still 1% of pairs misdiagnosed for LUAD and 1% for LUSC (Supplementary Table S4, available at https://doi.org/10.1016/j.esmoop.2025.105072). The median number of shared mutations between clonal tumor pairs increases with the size of the panel. The range of the number of matching mutations, however, were overlapping between clonal and non-clonal pairs (Supplementary Table S5, available at https://doi.org/10.1016/j.esmoop.2025.105072).

### In-depth evaluation of incorrectly classified tumor pairs

**Figure 3:**
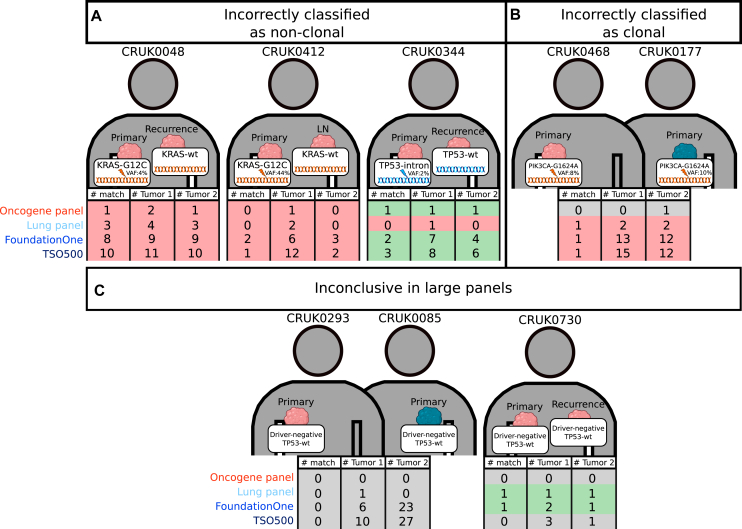
**Tumor pairs with incorrectly classified clonal relationships.** Three pairs of LUAD primary tumors and intrapulmonary metastasis were incorrectly classified as non-clonal (A) and one pair of interpatient LUSC tumors were incorrectly classified as clonal (B). For each tumor, the mutation status responsible for misclassification is indicated. Tumor pairs with an inconclusive result in any of the large panels are shown in the bottom panel (C). The number of matching mutations detected by each panel is indicated below each tumor pair, in addition to the number of total mutations detected in each tumor. Red shades indicate misclassifications, green shades indicate correct classifications and gray shades indicate an inconclusive result. Mutations and classification by each panel for all tumor pairs can be found in Supplementary Table S4, available at https://doi.org/10.1016/j.esmoop.2025.105072. LN, lymph node; LUAD, lung adenocarcinoma; LUSC, lung squamous cell carcinoma; VAF, variant allele frequency; wt, wildtype.

Two clonal LUAD tumor pairs were misclassified as non-clonal by all panels due to discordance in *KRAS* mutation status. The primary tumor had a *KRAS*-G12C mutation and the metastasis was *KRAS*-wildtype (Figure 3A, Supplementary Table S4A, available at https://doi.org/10.1016/j.esmoop.2025.105072). In one of these pairs, the variant allele frequency (VAF) of the detected *KRAS* mutation was low (3.7%) thus presumably a subclonal event. Although misclassified according to the IASLC clonality algorithm, this pair had a high number of matching mutations by all panels (Supplementary Table S4A, available at https://doi.org/10.1016/j.esmoop.2025.105072). In the other clonal pair, few matching mutations were detected between the primary (*KRAS* VAF 43%) and metastasis (*KRAS*-wildtype). Another clonal pair was misclassified due to a difference in *TP53* mutation status (primary: *TP53*-G422T intronic, metastasis: *TP53*-wildtype) only by the lung cancer panel (with full *TP53* coverage). The other panels correctly classified this pair (no intronic *TP53* regions covered), although few matching mutations were detected.

For LUSC, one interpatient tumor pair was misclassified as clonal for which identical *PIK3CA*-G1624A mutations were detected (Figure 3B). In large NGS panels many non-matching mutations were detected (Supplementary Table S4A, available at https://doi.org/10.1016/j.esmoop.2025.105072).

In one interpatient tumor pair no matching mutations were detected and therefore classified as inconclusive by all four panels (Figure 3C). Nevertheless, the large panels detected many non-matching mutations (Supplementary Table S4B, available at https://doi.org/10.1016/j.esmoop.2025.105072). One clonal pair was classified inconclusive by TSO500 and the oncogene panel since no matching mutations were detected.

## Discussion

In this study, we show that an oncogene panel is less suitable for clonality assessment, mainly due to a high number of inconclusive results. The NSCLC-specific panel, including commonly mutated tumor suppressor genes (with full coverage of TP53), and the two large panels have a much lower number of inconclusive results (1%-5%) and a high DTA (98%-99%). Nevertheless, all NGS panels consistently misclassified 1%-2% of pairs, mainly due to discordant presence of *KRAS-*G12C mutations or a coincidentally shared *PIK3CA* mutation.

Oncogenic driver mutations are considered to always occur as truncal mutations (i.e. present in all tumor cells) and are therefore critical in clonality assessment as acknowledged in the 2024 IASLC recommendations.^6^ In our data and the original TRACERx analyses, however, *KRAS* was found to occur subclonally in a minority of tumors.^7^^,^^8^ Interestingly, this was not observed in our TRACERx analysis for epidermal growth factor receptor (EGFR) or other oncogenic driver mutations. Hence, a mismatch in *KRAS* mutation status should be interpreted with caution for clonality assessment and in consideration with the genomic context (i.e. the VAF and other mutations). In addition, based on our findings, including *TP53* intronic mutations in the IASLC algorithm may lead to misclassification. When evaluating non-clonal tumor pairs, we observed two non-clonal tumors with an identical *PIK3CA* mutation which is reported to have a prevalence of 2.8% among LUSC tumors from The Cancer Genome Atlas.^10^ These results indicate that mutation prevalence should be taken into consideration when deciding on the clonality status of a tumor pair if little or no other mutations are detected, particularly if smaller panels are used.^11^ In large panels more mutations are detected, enabling more detailed and definitive interpretation of the clonal status when all mutations, also those in genes not part of the IASLC recommendation,^6^ are considered. For example, in the misclassified pairs highlighted in this study, different conclusions of clonality can be drawn from the large mutation panel tests. Finally, we also observed a minority of pairs which are inherently difficult to interpret based on any diagnostic NGS panel alone, presumably due to high subclonal diversity.^7^^,^^8^ These results underline the conclusion from the IASLC that next to mutation information, other information should be included to come to a definite decision on clonality.^6^^,^^12^ Undoubtedly, comprehensive evaluation of clinical, histological and multiple orthogonal sources of molecular information will lead to the optimal decision for clonality. Future studies including all these modalities in a real-world setting are warranted.

How should this study be interpreted in the light of daily molecular diagnostic practice? First of all, the cohort used for this study was not selected based on the clinical or histological dilemma of clonality assessment yet represents an average NSCLC population. In addition, we created interpatient pairs to complement the low number of intrapatient SPLC pairs, which may not necessarily reflect the same mutational scenario of two non-clonal tumors arising in one individual having one specific environmental exposure and hereditary susceptibility.^13^ From a technical perspective, the mutation data used for our study were obtained differently compared with daily molecular diagnostic practice. For our study, we used the mutation calls as presented by TRACERx that were derived from WES of fresh frozen tumors and, to subtract germline variants, patient-matched normal material. In clinical practice, however, mutation calls are commonly obtained with NGS panels using only tumor material that was formalin-fixed. Nevertheless, inherent differences in technologies (e.g. tumor-only, depth of coverage) have been evaluated in several previous studies showing that differences in pathogenic mutation calling between the various techniques have evolved to negligible differences, required for implementation in diagnostic practice.^14^^,^^15^ In small panels without matched normal, however, it remains challenging to differentiate somatic mutations from low prevalent germline variants.

**Figure 4:**
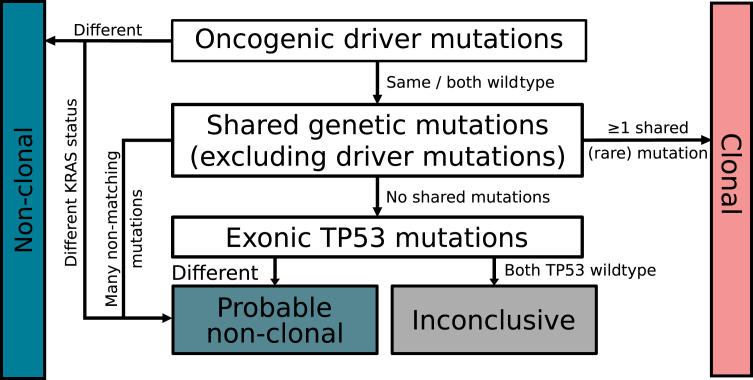
Recommended adaptations to the 2024 IASLC molecular classification algorithm. IASLC, International Association for the Study of Lung Cancer.

Discrepancies of *KRAS* mutation status should not prompt a definite non-clonal classification, especially when other detected mutations are shared. Given the prominent role of TP53 in the 2024 IASLC recommendations, only mismatches in exonic (rather than intronic) TP53 mutations should be included in the algorithm. Vice versa, a relatively high prevalent mutation can be coincidentally shared by non-clonal tumors. Generally, large NGS panels allow for interpretation of clonality using mutations in more genes than included in the IASLC recommendations (i.e. oncogenic drivers and *TP53*) and would correctly classify the majority of cases. Based on these observations, we propose minor adaptations to the IASLC molecular classification algorithm as displayed in Figure 4.

In conclusion, oncogene-only panels result in many inconclusive results. Adding tumor suppressor genes accurately resolves the majority of pairs according to IASLC recommendations. Nevertheless, for 1%-2% of patients clonality assessment is inherently difficult, which could lead to misclassification. For these cases, large NGS panels are preferred. Based on our findings, we recommend NGS panels, that preferably exceed 300 genes and an adapted IASLC algorithm.
