# Supplementary material for: Performance and considerations in the use of diagnostic mutation panels for clonality testing in non-small-cell lung carcinoma
Source: ESMO Open. 2025 May 14;10(5):105072. doi: 10.1016/j.esmoop.2025.105072 (PMC12141053; doi:10.1016/j.esmoop.2025.105072)
Supplement: Supplementary Material [file mmc6.docx]

**Supplementary Material**

**Supplementary Tables**

**Supplementary Table 1 (attached): Gold standard clonal and non-clonal tumor pairs created with samples from the TRACERx421 cohort.**

**Supplementary Table 2 (attached). Genomic regions of NGS mutation panels.** Genomic regions of Oncogene panel (a), lung panel (b), FoundationOne (c) and TSO500 (d).

**Supplementary Table 3. Performance of clonality classification by NGS mutation panels.** For incorrectly classified cases, the reason is indicated by letters as follows: *a*; Wildtype oncogenic driver and ≥ 1 shared mutations, *b*; Same oncogenic driver mutation and ≥ 1 shared mutations, *c*; Different oncogenic driver mutation, *d*; No shared oncogenic driver and different TP53 mutations, *e*; No shared oncogenic driver, no shared mutations and TP53 wildtype. FP, false positive (non-clonal case classified as clonal); FN, false negative (clonal case classified as non-clonal).

|  | **Percentage of correct clonal**  **pairs (sensitivity)** | **Percentage of correct non-clonal**  **pairs (specificity)** | **Inconclusive** | **Misclassified** | **Misclassifications** | | | | | |
| --- | --- | --- | --- | --- | --- | --- | --- | --- | --- | --- |
|  |  |  |  |  | **FP** | **Reason FP** | | **FN** | **Reason FN** | |
|  |  |  | ***e*** |  |  | ***a*** | ***b*** |  | ***c*** | ***d*** |
| **TSO500** |  |  |  |  |  |  |  |  |  |  |
| **LUAD** | 100%  (62/62) | 97%  (65/67) | 1%  (1/130) | 2%  (2/130) | 0 | 0 | 0 | 2 | 2 | 0 |
| **LUSC** | 98%  (40/41) | 100%  (38/38) | 1%  (1/80) | 1%  (1/80) | 1 | 1 | 0 | 0 | 0 | 0 |
| **FoundationOne** |  |  |  |  |  |  |  |  |  |  |
| **LUAD** | 100%  (63/63) | 97%  (65/67) | 0%  (0/130) | 2%  (2/130) | 0 | 0 | 0 | 2 | 2 | 0 |
| **LUSC** | 98%  (40/41) | 100%  (38/38) | 1%  (1/80) | 1%  (1/80) | 1 | 1 | 0 | 0 | 0 | 0 |
| **Lung panel** |  |  |  |  |  |  |  |  |  |  |
| **LUAD** | 100%  (56/56) | 96%  (65/68) | 5%  (6/130) | 2%  (3/130) | 0 | 0 | 0 | 3 | 2 | 1 |
| **LUSC** | 97%  (37/38) | 100%  (38/38) | 5%  (4/80) | 1%  (1/80) | 1 | 1 | 0 | 0 | 0 | 0 |
| **Oncogene panel** |  |  |  |  |  |  |  |  |  |  |
| **LUAD** | 100%  (35/35) | 96%  (54/56) | 30%  (39/130) | 2%  (2/130) | 0 | 0 | 0 | 2 | 2 | 0 |
| **LUSC** | 100%  (14/14) | 100%  (7/7) | 74%  (59/80) | 0%  (0/80) | 0 | 0 | 0 | 0 | 0 | 0 |

**Supplementary Table 4 (attached). Classification of tumor pairs by each NGS panel.** Mutations detected by each panel in misclassified (a), inconclusive (b) and correctly classified (c) tumor pairs.

**Supplementary Table 5. The median number of matching mutations overlap between clonal and non-clonal tumor pairs.** The median number of matching mutations is denoted with the range between parentheses.

|  | **Median number of matching mutations in clonal pairs (range)** | **Median number of matching mutations in non-clonal pairs (range)** |
| --- | --- | --- |
| **TSO500** | 13 (0-91) | 0 (0-1) |
| **FoundationOne** | 12 (1-67) | 0 (0-1) |
| **Lung panel** | 2 (0-5) | 0 (0-1) |
| **Oncogene panel** | 1 (0-4) | 0 (0-1) |

**Supplementary Methods**

**Samples and mutation data**

From the TRACERx421 cohort, we retrieved WES mutation calls for 1751 samples of 421 patients^1,2^. Patients with at least one tumor with confirmed LUAD or LUSC histology were selected, which resulted in 954 LUAD tumor samples from 244 patients and 587 LUSC tumor samples from 140 patients.

Clonal pairs were defined using one random sample taken from the primary lung tumor paired with one random sample from an intrapulmonary metastasis if available, leading to 60 intrapulmonary clonal pairs for LUAD and 38 intrapulmonary clonal pairs for LUSC. If no intrapulmonary metastasis pair was available, the primary tumor was matched with one randomly selected extrapulmonary metastasis sample, leading to 5 LUAD and 2 LUSC extrapulmonary metastasis for each patient.

Non-clonal pairs were defined using one random sample taken from the primary lung tumor paired with one random from an SPLC if available, leading to 7 pairs of SPLCs for LUAD and 0 for LUSC. To match the same amount of non-clonal and clonal pairs, additional non-clonal pairs were created by randomly matching primary tumors from different patients. This resulted in 58 interpatient tumor pairs for LUAD and 40 for LUSC. To summarize, the above procedure resulted in a total of 65 clonal and 65 non-clonal pairs for LUAD and 40 for LUSC.

Clonality of defined tumor pairs were verified by investigating the number of shared mutations detected by WES. Clonal pairs had a median Jaccard index of 71% (range 17%-97%) with a median of 468 shared mutations (range 51-4108). Non-clonal pairs had a median Jaccard index of 0% (range 0%-1%) with a median of 0 shared mutations (range 0-22). The maximum number of mutations shared between non-clonal pairs was 1 except for one pair of SPLCs which presented as a collision tumor (CRUK0881) with 22 matching mutations and a Jaccard index of 1.13%.

**In silico extraction of NGS mutations panel regions from WES mutation data**

WES mutation calls were downsampled to genomic regions representative of four common NGS panels. For downsampling we selected two small and two large NGS mutation panels, (I) The MayoComplete Lung Cancer-Targeted Gene Panel, an oncogene panel covering 0.034Mb across 12 genes (Mayo Clinic Laboratories, Rochester, MN), (II) A custom designed lung cancer-specific panel covering 0.016Mb across 22 commonly mutated genes (including full coverage of TP53, STK11 genes), and two large NGS mutation panels, (III) FoundationOne CDx (Foundation Medicine Inc., Cambridge, MA) and (IV) Illumina TruSight Oncology 500 (TSO500) (Illumina, San Diego, CA), covering 3.27Mb across 324 genes and 1.28Mb across over 523 genes respectively (Supplementary Table 2). NGS panel regions of the MayoComplete and TSO500 were retrieved in BED files format from the manufacturers. The lung cancer specific panel has been in routine clinical use at AmsterdamUMC. For FoundationOne CDx a publicly available descriptive table was retrieved^3^ to reconstruct genomic regions using GENCODE^4^ (v19) annotations.

**Objective evaluation of clonality assessment**

To assess clonality, mutations were compared between pairs of tumors using the molecular classification algorithm and procedures proposed by Yang et al^5^ and according to the IASLC 2024 guideline^6^. Here oncogenic driver mutations are defined as base pair transitions in EGFR, KRAS, BRAF and ERBB2 which are designated as ‘likely oncogenic’ or ‘oncogenic’ in the OncoKB database^7^ (v4.20). A known acquired therapy resistance mutation in EGFR (T790M) was excluded as a driver mutation. Tumor pairs with different oncogenic driver mutation status were classified as SPLCs. For tumor pairs with the same oncogenic driver status, additional mutations (excluding oncogenic driver mutations) were compared. Tumor pairs with at least one shared mutation were classified as metastasis. If no shared mutation was detected, TP53 mutation status was evaluated. If both tumors were TP53 wildtype, the pair was classified as inconclusive and if both tumors had a different TP53 mutation status, the pair was classified as SPLCs. This procedure was performed for all tumor pairs for the mutations found in each NGS panel separately.

For each NGS panel, the resulting clonality classification was subsequently compared with the gold-standard clonality. To evaluate the performance, we calculated the sensitivity (percentage of correct clonal predictions), the specificity (percentage of correct non-clonal predictions) and the diagnostic test accuracy (percentage of pairs correctly classified).

**References**

1. Al Bakir, M. *et al.* The evolution of non-small cell lung cancer metastases in TRACERx. *Nature* **616**, 534–542 (2023).

2. Frankell, A. M. *et al.* The evolution of lung cancer and impact of subclonal selection in TRACERx. *Nature* **616**, 525–533 (2023).

3. *FoundationOne ® CDx Technical Information*. https://info.foundationmedicine.com/hubfs/FMI%20Labels/FoundationOne_CDx_Label_Technical_Info.pdf.

4. Frankish, A. *et al.* GENCODE reference annotation for the human and mouse genomes. *Nucleic Acids Res* **47**, D766–D773 (2019).

5. Yang, C. Y. *et al.* Genomic Profiling With Large-Scale Next-Generation Sequencing Panels Distinguishes Separate Primary Lung Adenocarcinomas From Intrapulmonary Metastases. *Modern Pathology* **36**, (2023).

6. Chou, T.-Y. *et al.* Differentiating separate primary lung adenocarcinomas from intrapulmonary metastases with emphasis on pathological and molecular considerations: Recommendations from the IASLC Pathology Committee. *Journal of Thoracic Oncology* (2024) doi:10.1016/j.jtho.2024.11.016.

7. Chakravarty, D. *et al.* OncoKB: A Precision Oncology Knowledge Base. *JCO Precis Oncol* 1–16 (2017) doi:10.1200/PO.17.00011.
